# Desmin gene expression is not ubiquitous in all upper airway myofibers and the pattern differs between healthy and sleep apnea subjects

**DOI:** 10.1186/s40001-024-01812-9

**Published:** 2024-04-03

**Authors:** Per Stål, Hanna Nord, Jonas von Hofsten, Thorbjörn Holmlund, Farhan Shah

**Affiliations:** 1 Department of Medical and Translational Biology, Umeå University, SE-901 87 Umeå, Sweden; 2 Department of Clinical Sciences, Otorhinolaryngology, Umeå University, SE-901 87 Umeå, Sweden

**Keywords:** Desmin, mRNA, Cytoskeleton, Snoring, Obstructive sleep apnea, Muscle fiber injury, Vibration

## Abstract

**Background:**

Desmin is a major cytoskeletal protein considered ubiquitous in mature muscle fibers. However, we earlier reported that a subgroup of muscle fibers in the soft palate of healthy subjects and obstructive sleep apnea patients (OSA) lacked immunoexpression for desmin. This raised the question of whether these fibers also lack messenger ribonucleic acid (mRNA) for desmin and can be considered a novel fiber phenotype. Moreover, some fibers in the OSA patients had an abnormal distribution and aggregates of desmin. Thus, the aim of the study was to investigate if these desmin protein abnormalities are also reflected in the expression of desmin mRNA in an upper airway muscle of healthy subjects and OSA patients.

**Methods:**

Muscle biopsies from the musculus uvulae in the soft palate were obtained from ten healthy male subjects and six male patients with OSA. Overnight sleep apnea registrations were done for all participants. Immunohistochemistry, *in-situ* hybridization, and reverse transcription–quantitative polymerase chain reaction (RT–qPCR) techniques were used to evaluate the presence of desmin protein and its mRNA.

**Results:**

Our findings demonstrated that a group of muscle fibers lacked expression for desmin mRNA and desmin protein in healthy individuals and OSA patients (12.0 ± 5.6% vs. 23.1 ± 10.8%, *p* = 0.03). A subpopulation of these fibers displayed a weak subsarcolemmal rim of desmin accompanied by a few scattered mRNA dots in the cytoplasm. The muscles of OSA patients also differed from healthy subjects by exhibiting muscle fibers with reorganized or accumulated aggregates of desmin protein (14.5 ± 6.5%). In these abnormal fibers, the density of mRNA was generally low or concentrated in specific regions. The overall quantification of desmin mRNA by RT–qPCR was significantly upregulated in OSA patients compared to healthy subjects (*p* = 0.01).

**Conclusions:**

Our study shows evidence that muscle fibers in the human soft palate lack both mRNA and protein for desmin. This indicates a novel cytoskeletal structure and challenges the ubiquity of desmin in muscle fibers. Moreover, the observation of reorganized or accumulated aggregates of desmin mRNA and desmin protein in OSA patients suggests a disturbance in the transcription and translation process in the fibers of the patients.

## Background

We have previously reported the absence of the cytoskeletal protein desmin in a subgroup of muscle fibers in the soft palate of healthy individuals and patients suffering from obstructive sleep apnea (OSA) [1–3]. However, no studies have reported whether mRNA for desmin is also absent in these fibers.

Desmin is a muscle-specific intermediate filament (IF) considered to be ubiquitous in all muscle fibers. This IF protein plays a crucial role in muscle function as it forms a three-dimensional scaffold throughout mature muscle fibers, providing structural support, mechanical integrity, force transmission, and load bearing [4, 5]. Seminal research during the last decades has revealed that desmin IF also influences various biological processes, including myogenesis [6], mitochondrial function [7–9] and homeostasis in atrophying and diseased muscle [10]. Mutations in desmin cause myofibrillar misalignment, mitochondrial dysfunction, and impaired mechanical integrity leading to myopathies, often characterized by the accumulation of protein aggregates [4, 10, 11].

Desmin is encoded by a single gene and is among the earliest proteins that appear during myogenesis [7, 12]. Given its critical role in muscle function and its ubiquity, the absence of desmin in the upper airway muscles of healthy subjects raises intriguing questions about the existence of a novel fiber phenotype with unique cytoarchitecture [1].

Our earlier findings of disorganization and accumulation of aggregates of desmin in soft palate muscles of obstructive sleep apnea (OSA) patients indicate muscle injury and the occurrence of pre- and post-translational modifications in the protein synthesis [2, 3, 13]. These alterations align with the morphological and pathological changes reflecting acquired neuropathy and myopathy reported in the upper airway muscles of OSA patients [2, 3, 13–17]. The cause of these alterations is largely unclear, but traumatic snoring vibrations have been suggested to be a potential cause for neuromuscular injuries and pharyngeal dysfunction in OSA patients [2, 3, 13].

A normal expression of desmin protein in a muscle requires the presence of mRNA, a functional protein translation, and a ubiquitin–proteasome system [18, 19]. Our objective was to investigate whether muscle fibers in the soft palate lacking immunoexpression of desmin protein also lack desmin mRNA in healthy humans. In addition, we aimed to examine the mRNA expression pattern in pathological muscle fibers from OSA patients where the distribution of desmin was reported to be disrupted.

## Methods

### Healthy subjects and OSA patients

The patient group was selected from subjects referred with complaints of snoring and OSA. Exclusion criteria for both groups were any significant disease, previous surgery in the oropharynx, medications, smoking, and drug abuse. Additional exclusion criteria for the voluntary healthy subjects were snoring and OSA.

### Clinical investigation and sleep apnea recordings

All subjects underwent similar clinical examinations. A portable ambulatory overnight sleep apnea device (Embletta, Embla Systems, Kanata, Canada) was used (Type 3 study). The device recorded continuous signals for airflow using nasal cannula pressure, thoracic and abdominal respiratory movements, recording of snoring sounds, finger pulse-oximetry (Nonin Oximeter Xpod, Nonin Medical Inc. Plymouth), and body position. The recordings, which was performed for a minimum of 4 h during sleep (average sleeping time 6.3 h for patients and 6.6 h for healthy subjects), were manually scored according to the American Academy of Sleep Medicine recommendations. An apnea was defined as a ≥ 90% cessation of airflow lasting at least 10 s, while a hypopnea was defined as a 50% reduction in airflow compared with baseline, combined with an oxygen desaturation of ≥ 3% [20].

### Clinical outcome

The criteria for the healthy subject group were fulfilled by six subjects, all males, mean age 38 years (range 30–49) and a mean body mass index (BMI) of 24 kg/m^2^ (range 22–26). Overnight sleep registration revealed that none of them snored or had OSA.

In the patient group, snoring and OSA were confirmed by overnight sleep registration in eight male patients; the mean age was 46 years (range 29–77), and BMI was 30 kg/m^2^ (range 26–34). The mean AHI (apnea–hypopnea index) was 13.5. The subjects were referred for upper-airway surgery because of snoring and OSA.

### Muscle samples

The biopsies in healthy subjects were obtained from the base of the uvula with punch biopsy technique (4 mm biopsy punch, Miltex, Inc, USA), except for one subject where complete surgical resection of the uvula was performed due to large size and discomfort. In patients, the entire base of the uvula was resected in connection with soft-palate surgery. Specimens from the middle part of musculus uvula were mounted in OCT (Tissue Tek, Miles, Elkhart, IN, USA) compound and snap-frozen in liquid propane chilled with liquid nitrogen. All samples were stored at – 80 °C until further processing.

### Staining for basic histology

The muscle samples were cut at – 20 °C into 8–10 μm thick cross-sections in a cryostat (Leica CM 3050, Nussloch, Germany), and the sections were mounted on glass slides. For a demonstration of basic morphology, the samples were stained with routine hematoxylin–eosin (H&E).

### *In-situ* hybridization and immunohistochemistry

*In-situ* hybridization was performed on muscle cross-sections using Riboprobe targeting desmin mRNA (BC032116, BioScience UK) according to a protocol previously described [21]. For visualization of mRNA, Fast Red TR/Naphthol S-MX and TRIS tablets (SIGMA-ALDRICH Co, Spruce Street, St Louis, USA) were used.

Immediately after *in-situ* hybridization, immunohistochemical staining with a well‐characterized polyclonal antibody (pAb) (15200) against desmin (DES) was performed in the same section. This pAb has previously been shown to mark amino acid sequence 400 in the c-terminus of the DES molecule [22]. To confirm the expression pattern of the DES protein in muscle fibers, two separate sections were immunostained with either monoclonal antibody (mAb) M0760 or mAb18-0016 directed against DES. In these sections, the basement membrane (i.e., basal lamina) of muscle fibers was visualized using laminin pAb PC128. For details of the antibodies, see Table 1. In short, the sections were immersed in 5% normal non‐immune donkey serum (Jackson ImmunoResearch Laboratories, West Grove, PA, USA) for 15 min and rinsed in 0.01 M phosphate‐buffered saline (PBS) for 3 × 5 min. The sections were then incubated for 2 h at 4 °C with the primary antibody diluted to appropriate concentrations in PBS with bovine serum albumin in a humid environment. After additional washes in PBS, the sections were incubated with the secondary Ab (37 °C for 30 min) and washed in PBS for 3 × 5 min. The bounded primary Abs were visualized by indirect immunofluorescence using affinity‐purified Abs prepared for multiple labeling conjugated with fluorochrome with different emission spectra; fluorescein isothiocyanate (FITC), Alexa fluor 488 and 647 (Invitrogen, CA, USA). Thereafter the samples were washed in PBS for 3 × 5 min, and a mounting medium with DAPI (4′, 6-diamidino-2-phenylindole dihydrochloride) was used to visualize nuclei (H-1500, Vector Lab, Burlingame, CA, USA). For control, serial cross-sections were treated as described above, with the exception that the primary antibody was exchanged with non-immune serum.

**Table 1:** Antibodies used for immunohistochemistry

Antibody	Product code	Gene*	Host/clone	Dilution	Source
Desmin	M0760	*DES*	mAb-mouse/D33	1:100	1
Desmin	18-0016	*DES*	mAb-mouse/ZC18	1:1000	2
Desmin (c-terminus)	ab15200	*DES*	pAb-rabbit	1:2000	3
Laminin	PC 128	*LAM*	pAb-sheep	1:15000	4

*Official gene nomenclature according to OMIM. (http://www.ncbi.nlm.nih.gov/omim/). 1. Dako, Sweden, 2. Invitrogen Corporation, CA, USA, 3. Abcam, UK, 4. Binding site Inc, USA

### Analysis and quantification

To obtain a minimum of 250 fibers from each case, two to four random areas of each muscle cross-section were scanned at 20 × magnification with a fluorescence microscope (Leica DM6000B; Leica Microsystems CMS GmbH, Wetzlar, Germany) equipped with a digital high‐speed fluorescence CCD camera (Leica DFC360 FX). Quantification of fibers either lacking or having maldistribution of desmin and or mRNA for desmin was manually performed on the scanned sections using the count tool of the Photoshop software (CS5, version 12.0.4, San Jose, CA, USA).

### Gene expression using real-time quantitative reverse transcription PCR (RT–qPCR)

Unfixed muscle samples were homogenized in QIAzol (Qiagen; #79306) using a handheld TissueRuptor (Qiagen). The homogenate was placed on the benchtop for 5 min to promote dissociation of nucleoprotein complexes. Chloroform was then added to the tube (ratio of 1:5) and shaken vigorously for approximately 15 s. The homogenate was centrifuged at 18 600 × *g* at 4 °C for 15 min, after which the upper aqueous phase was transferred to a new tube and mixed with 1.5 volume of 100% ethanol. Total RNA was extracted using the RNeasy Mini Kit (Qiagen; #74106); cDNA was synthesized using the High-Capacity cDNA Reverse Transcription kit (Applied Biosystems; #4268813). qRT-PCR was performed with TaqMan human probes for DES (#Hs00157258). The amplification was performed on a ViiA 7 Real-time PCR system (Applied Biosystems). Thermal-cycling conditions were 50 °C for 2 min, 95 °C for 20 s, 40 cycles of 95 °C for 1 s, and 60 °C for 20 s. Data were analyzed with ViiA 7 software (Applied Biosystems). The expression was normalized to β-actin levels (Applied Biosystems; #Rn00821946).

### Statistical analyses

Statistical analyses were performed using statistical software SPSS (version 23, IBM Corporation, USA). Due to small sample size, bootstrap statistics was used. The groups were compared by independent samples *t*-test. *p* ≤ 0.05 was considered significant.

### Ethical clearance

The regional Medical Ethical Committee in Umeå approved the study (Dnr-05-130 M). The voluntary healthy subjects and patients were informed and gave their written consent to participate. All muscle samples were collected in agreement with the Declaration of Helsinki.

## Results

### General muscle morphology

**Fig. 1:**
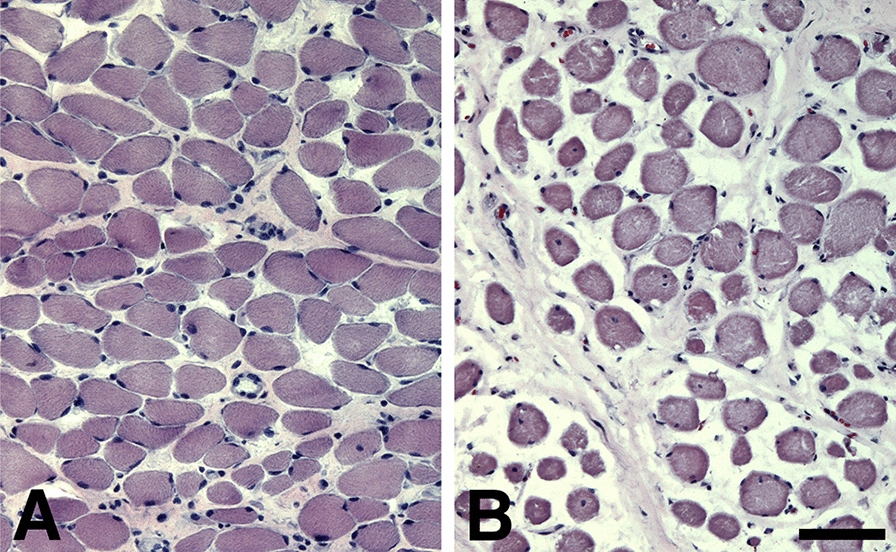
Overview of muscle cross-sections from the musculus uvula stained for H&E in a healthy individual **A** and a patient **B**. Note the high fiber size variability and high amount of connective tissue in the patient muscle. Scale bar = 50μm

**Fig. 2:**
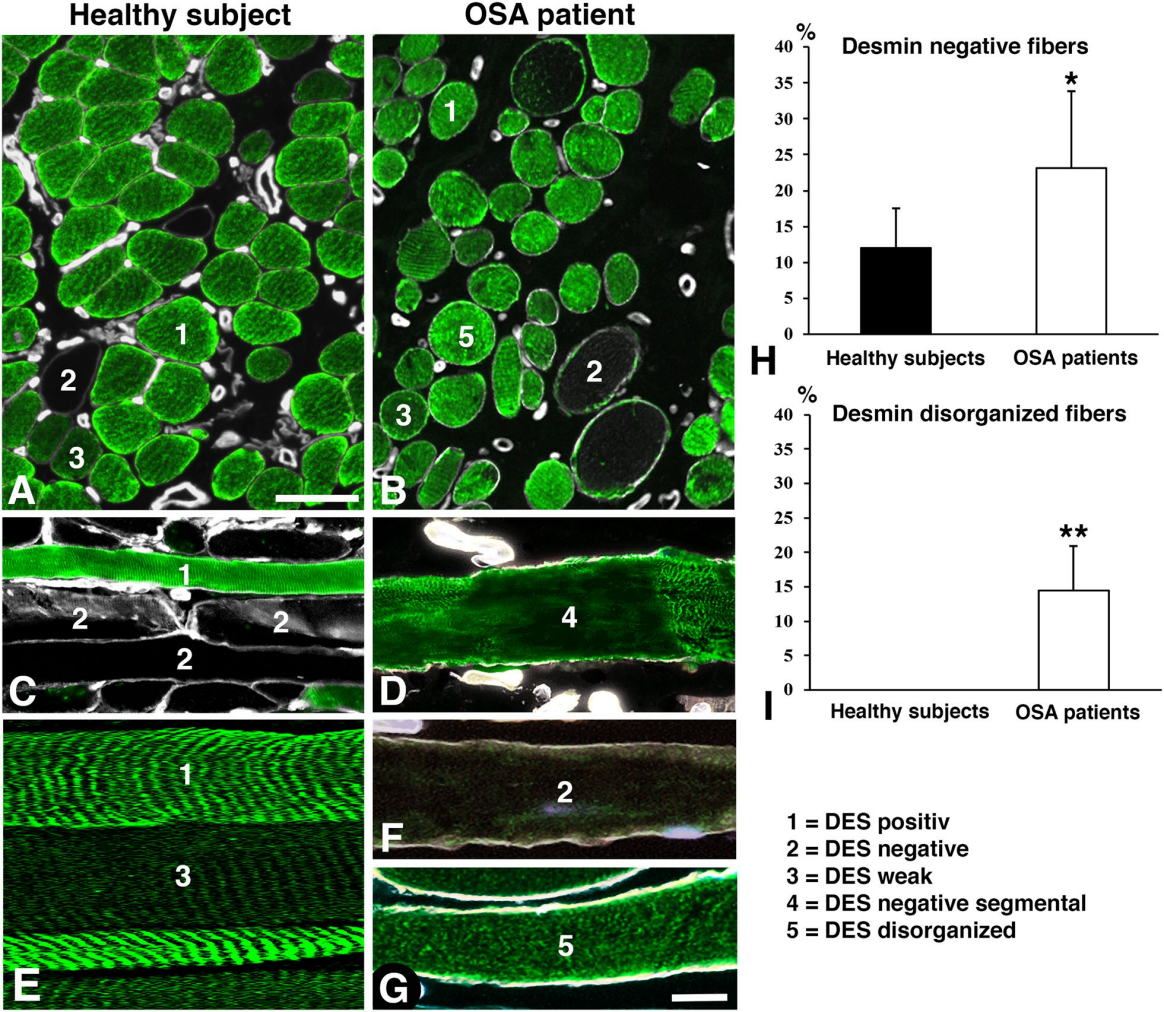
Cross- (**A**, **B**) and longitudinal sections (**C**–**G**) from the uvula muscle of healthy subjects (**A**) and an OSA patient immunostained for desmin (DES, green) and laminin (white, except E). Bar graphs show the percentage of desmin negative fibers (**H**) and disorganized fibers (**I**) in healthy subjects and patients (* *p*<0.05, ** *p*<0.01). Muscle fibers showing typical positive DES expression are marked 1, fibers lacking DES are marked 2, and fibers with weak DES staining are marked 3. In the OSA patient, muscle fibers with segmental lack of DES are marked 4 and fibers with disorganized DES are marked 5. Note the higher variability in fiber size, connective tissue content, and irregularity in the distribution of DES in fibers from the patient compared to the healthy subjects. Note also the lower capillarization in the sample from the patient. Scale bar panels **A**–**C**=50μm, panels **D**–**G** = 25μm

**Fig. 3:**
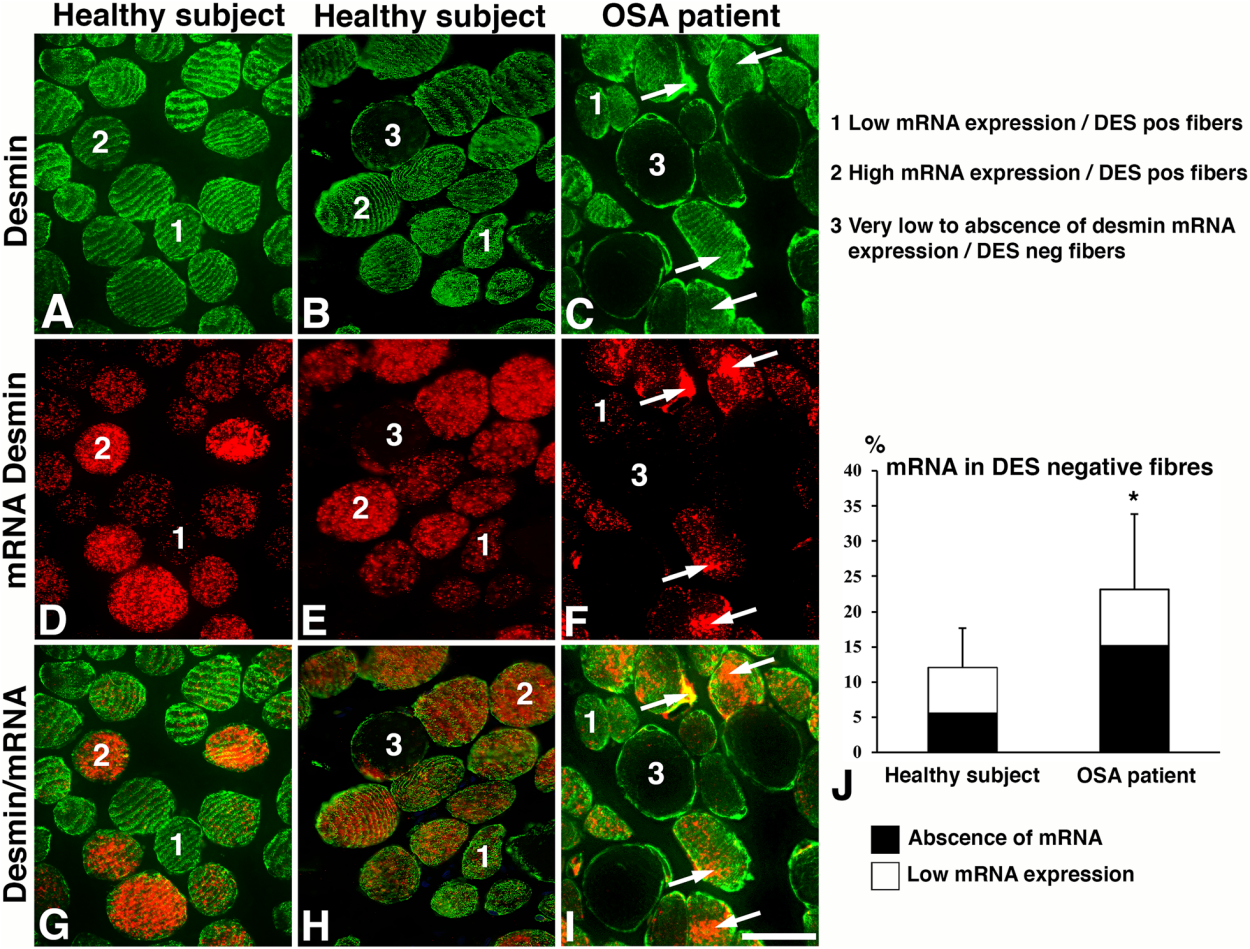
Cross-sections from the uvula muscle showing different regions in a healthy subject (columns 1 and 2) and an OSA patient (column 3) immunostained for desmin (DES, green, **A**–**C**) and a riboprobe against desmin mRNA (red, **D**–**F**). Merged staining for DES and desmin mRNA are shown in panels **G**–**I**. Muscle fibers displaying either low or high mRNA expression in DES positive fibers (DES pos) are marked 1 and 2, respectively. Fibers lacking desmin (DES neg) and its mRNA are marked 3. Bar graph (**J**) shows the percentage of DES neg fibers with absence or very low mRNA expression in healthy subjects and patients (p=0.05). Note the intra-myofibrillar maldistribution and accumulations of mRNA (arrows) as well as the higher number of fibers lacking expression for DES and its mRNA in OSA patients. Scale bar=50μm

**Fig. 4:**
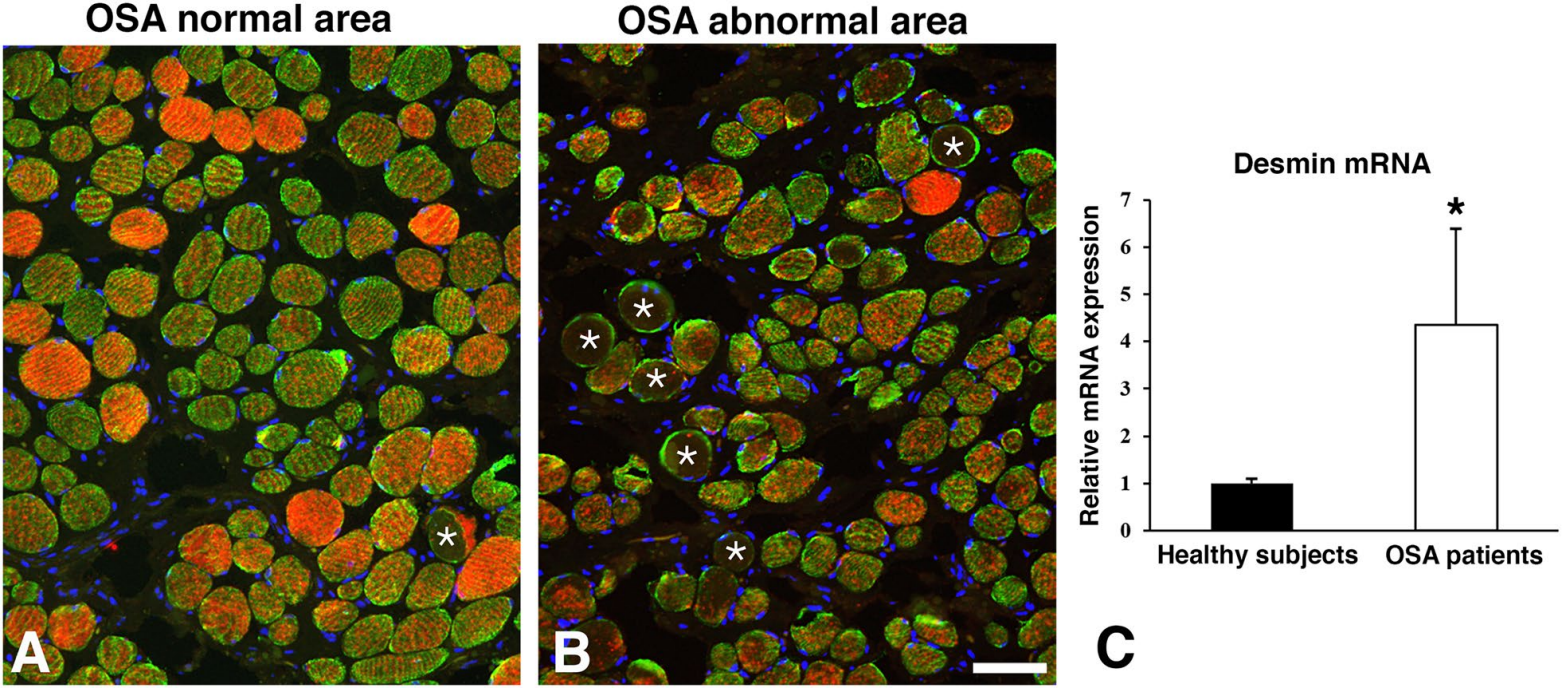
Cross-sections from different areas of the uvula muscle of an OSA patient stained for desmin (DES, green), its corresponding mRNA (red), and DAPI (nuclei blue). Panel **A** show an area in the muscle with a relatively normal morphology, while panel **B** shows an area with an abnormal appearance. In panel **B**, note the high variability in fiber size and fiber form, the presence of fibrosis, the generally lower expression of mRNA, and the presence of fibers lacking immunostaining for both DES and its mRNA (asterisks). Scale bar=50μm. In panel **C**, the bar graph shows significantly higher expression values of desmin mRNA (*) in the OSA patients than in the healthy subjects, as revealed by RT-qPCR

**Fig. 5:**
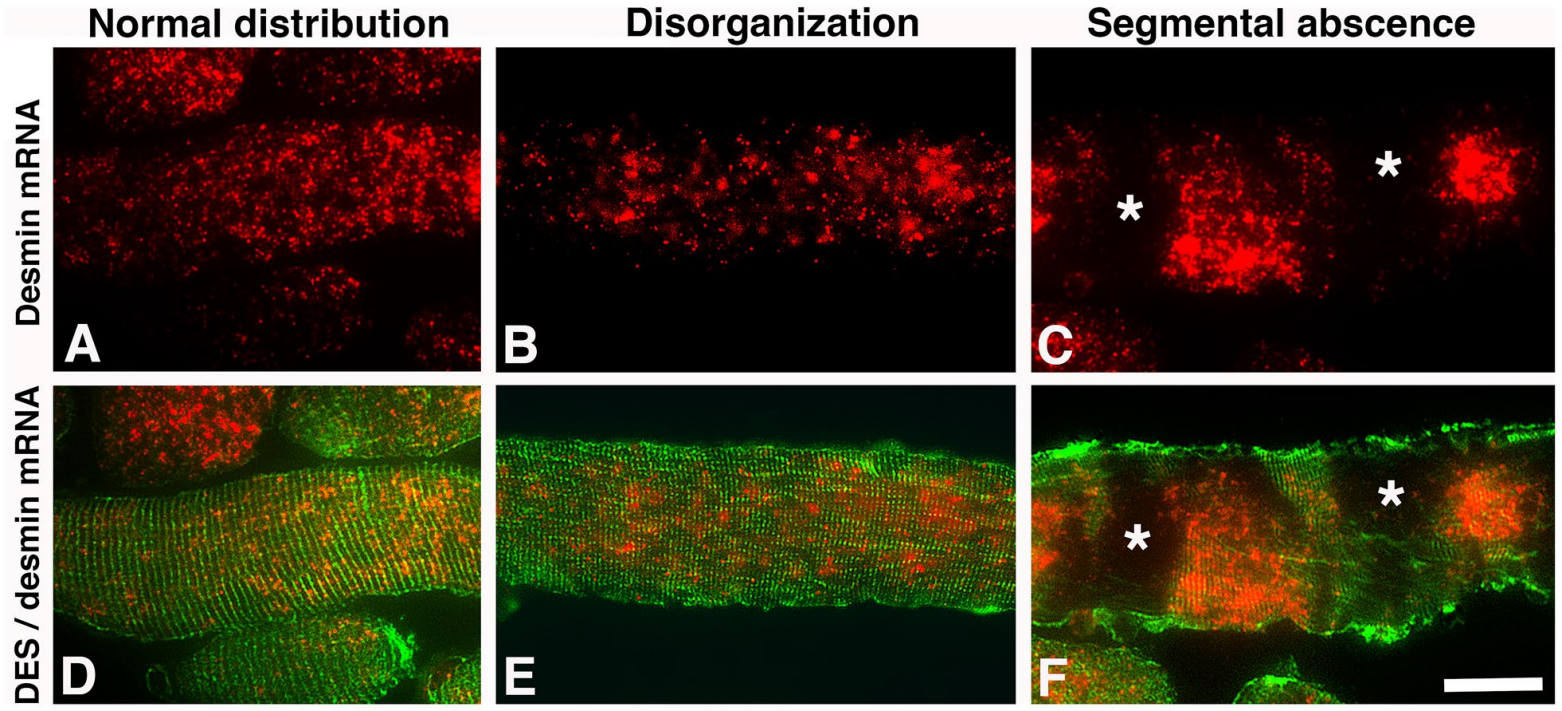
Longitudinally sectioned muscle fibers from a patient showing the expression of desmin mRNA (**A**–**C**, red) and merged expression of desmin mRNA and desmin (DES) protein (red and green, respectively, **D**–**F**). **A** and **D** show a fiber with a presence of desmin mRNA in a typical striated pattern of DES. **B** and **E** show a fiber with a more irregular distribution of both mRNA and DES. **C** and **F** show a fiber with a segmental lack of immunoreaction for DES and its mRNA. Note that mRNA and the protein for desmin also are maldistributed and accumulated in these fibers (**C** and **F**). Scale bar = 25 µm

**Fig. 6:**
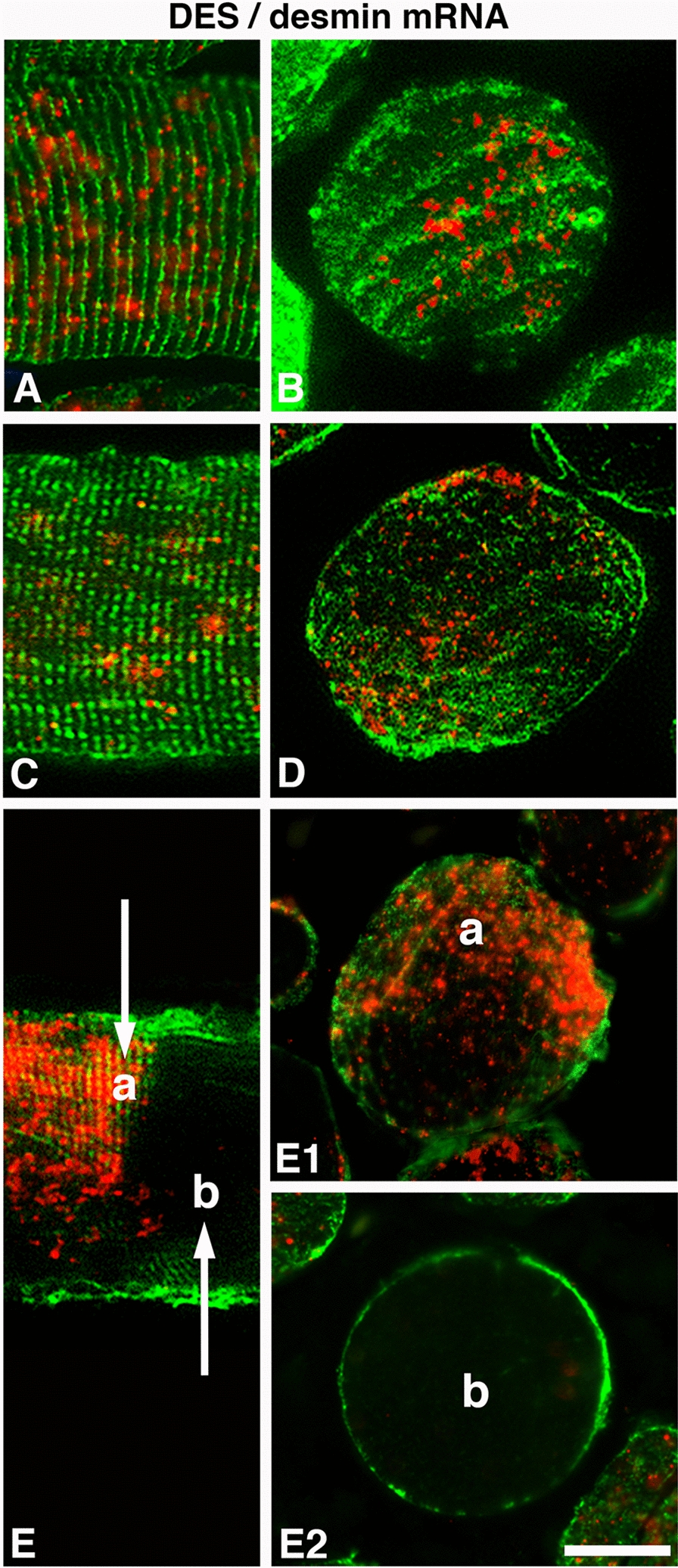
The figures illustrate the co-expression of desmin (DES, green) and desmin mRNA (red) in longitudinal (**A**, **C**, **E**) and cross-sectioned (**B**, **D**, **E**1 and **E**2) muscle fibers from an OSA patient. **A** and **B** show the expression pattern of DES and its mRNA in a normal fiber, and **C** and **D** show the expression pattern of mRNA in a fiber with disorganized DES. **E** reveals the staining pattern in a longitudinally sectioned fiber segmentally lacking DES and its mRNA. Note the accumulation of desmin mRNA in the area showing expression of DES (**E** and** E1**, marked **a**) and the lack of mRNA in the area lacking DES (**E** and **E2**, marked **b**). Scale bar = 25 µm

**Fig. 7:**
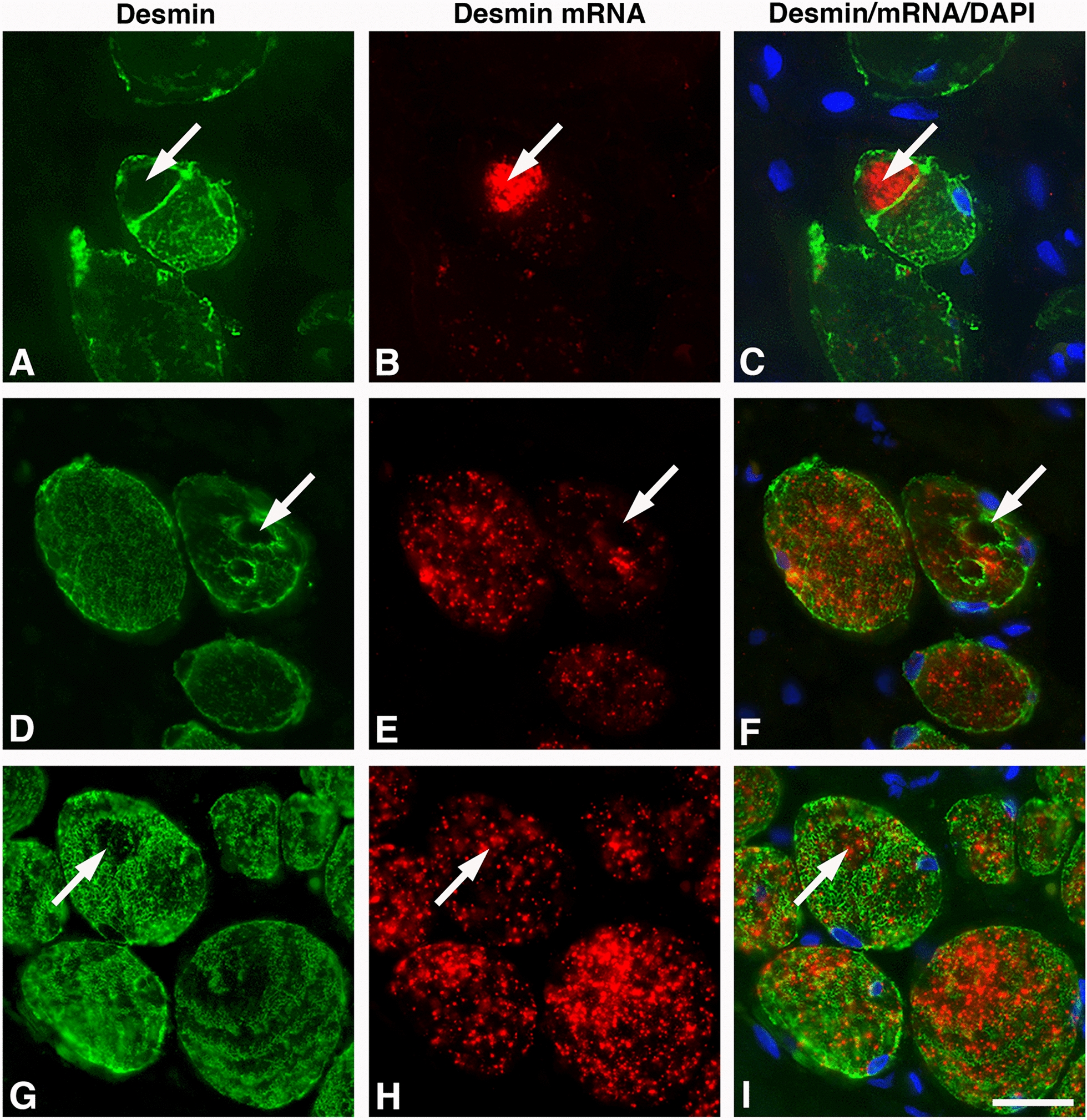
Muscle cross-sections from an OSA patient stained for desmin (DES, green, **A**, **D**, **G**), desmin mRNA (red, **B**, **E**, **H**), and merged staining with DAPI (nuclei blue) (**C**, **F**, **I**). **A**–**C** show a fiber with a region lacking DES but having a dense immunoreaction for its mRNA (arrows). **D**–**F** show a vacuole-like structure lacking immunostaining for DES and its mRNA (arrows). **G**–**I** show a circular area within the fiber having a very low expression of the DES but normal expression of desmin mRNA (arrows). Scale bar = 25 µm

**Fig. 8:**
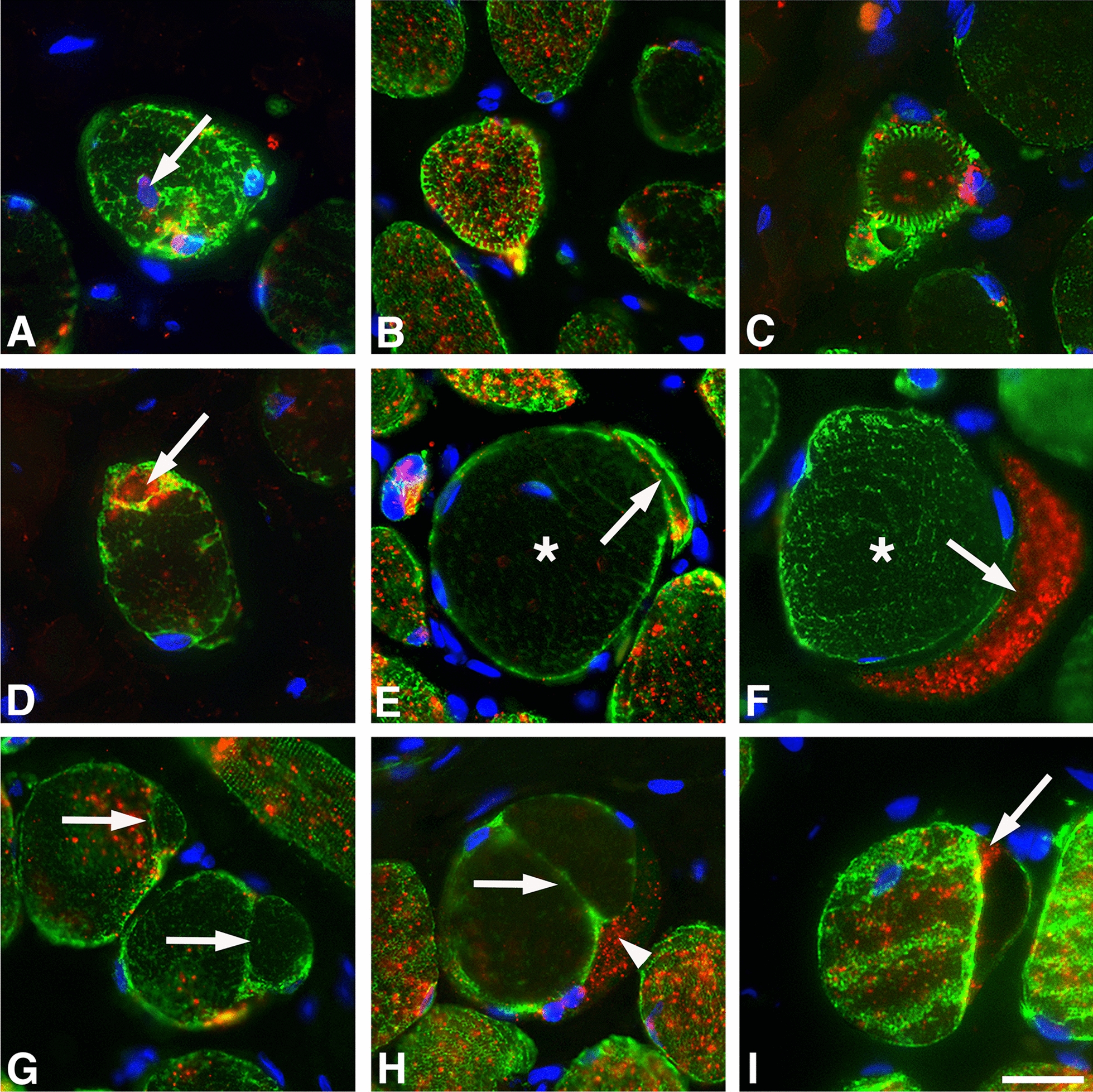
Cross-sections of muscle fibers displaying an abnormal expression of desmin protein and corresponding mRNA in OSA patients (**A**–**I**). The sections are stained for the desmin protein (DES, green), desmin mRNA (red), and DAPI (nuclei blue). Panel **A** shows a fiber with disorganized DES, very low expression of desmin mRNA, and an internal nuclei (arrow). Ringed fibers showing different patterns of maldistributed DES and corresponding mRNA are shown in panels **B** and **C**. Panel **D** shows a fiber with very weak staining for DES and a focal subsarcolemmal area filled with desmin mRNA (arrow). Panels **E** and **F** show a slender fiber-like structure (arrows) in close connection with a large fiber lacking or having a low amount of DES (asterisks). Note the very low expression of desmin mRNA in the slender fiber in panel **E** and very dense expression in the fiber in panel **F**. Panels **G** and **H** shows split fibers (arrows) almost entirely lacking both DES and its mRNA. Note in panel **H** that the fiber has an extension filled with desmin mRNA (arrowhead). Panel **I** show a fiber having a large extension lacking DES with a small subsarcolemmal accumulation of desmin mRNA (arrow). Scale bar=25μm

The muscles of the healthy subjects showed a muscle morphology as previously reported for healthy subjects [1, 23] (Figs. 1–3). In contrast, the muscles from the OSA patients displayed fibers with various morphological and pathological changes reflecting acquired neuropathy and myopathy [3, 13] (Figs. 1–8). No noticeable difference in morphology and pathology were observed between the 77 year-old patient and the other subjects in the patient group (mean age 41 years, range 29–50).

### Desmin protein expression in muscle fibers

In the healthy subjects, the majority of muscle fibers had an immunostaining pattern, as typically observed for DES in healthy muscles. However, a subgroup of muscle fibers lacked a distinct immunoreaction for DES (12.0 ± 5.6%). The expression pattern ranged from a complete lack of staining to fibers having a thin rim of subsarcolemmal staining. In general, the size and form of these fibers were similar to the fibers displaying a normal expression of desmin (Figs. 2–3).

In contrast, the muscle fibers from OSA patients differed by having three major immunostaining patterns for desmin; (1) muscle fibers with a normal staining pattern; (2) fibers lacking or only having a weak, thin rim of subsarcolemmal staining; (3) fibers with disorganized desmin or small to large intramyofibrillar aggregates of desmin (Fig. 2). In longitudinal sections, the fibers with absence or maldistribution of DES were occasionally observed in segments along the myofibril (Figs. 2, 5 and 6). This pattern was not observed in the longitudinally sectioned samples from healthy subjects. However, it cannot be ruled out that there are some segmental variations even in controls as the entire length of the fiber was not analyzed due to technical reasons. The proportion of muscle fibers with an absence of DES was significantly higher in OSA patients than in healthy subjects (23.1 ± 10.8%, p = 0.03), and muscle fibers with disorganization or aggregates of DES were observed in 14.5 ± 6.5% of the fiber population (Figs. 2 and 3). Some fibers had vacuole-like structures or restricted areas that were completely empty of DES or showed a rubbed-out pattern (Fig. 7).

### Desmin mRNA expression in relation to desmin protein

#### Desmin mRNA in muscle fibers with a normal expression of desmin protein

In both healthy and OSA subjects, the muscle fibers with a normal immunostaining pattern for DES generally showed an evenly spread-out expression of desmin mRNA throughout the muscle fiber cross-section. These fibers displayed generally a moderate density of mRNA but fibers with a low or high expression was also observed. The variability in the expression of desmin mRNA was higher in the muscles of OSA patients compared to healthy subjects (Figs. 3 and 4).

#### Desmin mRNA in muscle fibers lacking desmin protein expression

In the healthy subjects, out of the 12.0% of fibers showing absence of DES, 5.6 ± 1.3% completely lacked desmin mRNA, and 6.2 ± 3.7% displayed some few evenly spread out dots of mRNA in the cytoplasm (Fig. 3). A strong expression for desmin mRNA was seen in 0.2 ± 0.1% of the desmin empty fibers. The corresponding values in OSA patients showed that out of the 23.1% of fibers lacking DES, 15.2 ± 2.9% lacked or had only a few stained desmin mRNA dots, and 7.6 ± 2.9% displayed even spread-out dots of varying density (Fig. 3). Most of the fibers with areas devoid of DES in the cytoplasm lacked or had a very low mRNA staining in these areas. However, a few of the fibers lacking DES in certain areas showed a very strong mRNA expression in the DES empty zones (Fig. 7). In the longitudinal sectioned fibers of OSA patients, the areas lacking DES in certain segments along the fiber also lacked expression for desmin mRNA (Figs 2, 5 and 6).

#### Desmin mRNA in muscle fibers of patients with a maldistributed desmin protein

Of the 14.5% of fibers with disorganization or aggregation of DES, 9.9 ± 2.4% also displayed mRNA for desmin in a maldistributed or aggregated form (Figs. 4–8). The remaining 4.6 ± 1.0% of fibers showed a very low to almost complete absence of mRNA expression. In the fibers where the myofibrils were reorganized peripherally in the right angle to normal orientation, i.e., ring fibers, the mRNA for desmin varied from relatively high density to nearly total absence of expression (Fig. 8).

### Gene expression qRT-PCR

To further investigate the overall expression of desmin mRNA in the biopsies from healthy subjects and patients, a qRT-PCR analysis was performed. Desmin mRNA expression was significantly upregulated (*p* = 0.01) in patients compared to healthy subjects (Fig. 4C).

## Discussion

This study shows novel evidence indicating muscle fibers with a unique cytoarchitecture in the human soft palate. Our result revealed that a subpopulation of fibers in the musculus uvulae lacks muscle-specific cytoskeletal protein desmin and its mRNA. Previously we have shown that both the adult and infant palatopharyngeus muscle of healthy subjects also display fibers lacking desmin [1]. These findings challenge the prevailing notion that desmin is ubiquitous in all human muscle fibers and suggests the existence of a specialized subtype of muscle fibers in the soft palate. Interestingly, our investigation also revealed significant differences in the staining pattern for desmin and its transcript in muscle samples obtained from OSA patients compared to healthy subjects. Not only did the OSA patients exhibit a higher proportion of fibers lacking desmin and its mRNA, but their muscle fibers also displayed disorganization and aggregates of both the protein and mRNA for desmin.

The normal expression of the desmin protein in muscle fibers depends on the presence of mRNA and an adequate translation of the protein in the cell. Moreover, the variability in the amount or location of mRNA relies on the protein turnover and the need for the rate at which mRNA translates to protein in the cell [24]. This process involves a delicate balance between the renewal or replacement of degrading proteins, and the relationship between protein turnover and mRNA expression in the fibers may therefore vary. This might explain the various staining patterns for desmin protein and mRNA in the muscle fibers of the soft palate. It is worth noting that no previous studies have reported conditions in which desmin is completely inhibited at both the mRNA and protein levels. Thus, we propose that the desmin-empty fibers in the musculus uvula of healthy subjects represent a unique fiber phenotype with distinct properties. This is supported by our previous observation showing that the desmin empty muscle fibers also contained a C-terminal truncated dystrophin in the plasma membrane [2]. Dystrophin is a vital part of a protein complex connecting the muscle fiber cytoskeletal proteins, including desmin, and it transfers the forces from the contractile apparatus to the extracellular matrix. Normally, the dystrophin C-terminus binds to the multi-protein dystrophin–glycoprotein complex in the cell membrane - (DGC). To our knowledge, C-terminal truncated dystrophin has only been reported in a Duchenne muscular dystrophy phenotype where a nonsense codon mutation in the transcripted mRNA led to a truncated protein isoform [25, 26]. Considering the structural and functional link between desmin filaments and the DGC [10], the absence of both desmin and a typical dystrophin C-terminus further supports the presence of a structurally and functionally specialized fiber phenotype. Conducting future studies involving single-cell genome sequencing on these novel muscle fibers can provide further insights into their protein and gene composition.

The background behind the unique fibers in the human soft palate is unclear. However, it could relate to a specialization to meet the specific demands of various complex oropharyngeal tasks as shown earlier for human jaw muscles [27, 28]. The soft palate has an intricate muscle anatomy where five pairs of muscles act against each other in different directions to perform complex functions such as respiration, speech, swallowing, and ventilation of the ear. During contractions that involve elevation, depression, shortening, or stretching of the soft palate, the tense forces exerted by each muscle act obliquely against the fiber direction of the other muscles. In addition, these muscles lack a firm attachment at one or both ends. Considering this intricate muscle anatomy, a unique cytoskeletal protein complex might better fulfill functional demands by influencing fiber stiffness, sarcolemmal deformability, costamere stability, contraction velocity, and force transmission to the extracellular matrix.

In OSA patients, the higher proportion of muscle fibers with an absence of desmin and its transcript, along with the presence of disorganized and aggregated desmin and mRNA, is of particular interest. Both disorganization and aggregates of desmin have previously been described in several myopathies [19, 29, 30], but to our knowledge, no disease has been related to the absence of desmin and its mRNA in muscle fibers. The finding of a significantly higher number of desmin empty fibers in the muscle cross-sections of patients probably relates to the fact that some desmin-positive fibers in patients also displayed segmental loss of desmin (Figs. 2 and 5). Since this pattern was only observed in patients, this most likely indicates muscle damage. However, as we did not analyze the entire length of the fibers due to the variable fiber orientation, we could not exclude that there can be some variability in expression of desmin or other cytoskeletal and contractile proteins along the fibers also in the healthy individuals. Variation in contractile proteins along the fibers have been reported in animals [31].

The disorganization and accumulation of aggregates of desmin protein and its mRNA in myofibrils of OSA patients provide further evidence for myofibrillar injuries. In the desmin maldistributed fibers, the expression pattern for the desmin mRNA varied greatly between and within fibers. Even in fibers with a typical immunostaining pattern for the desmin protein, the variability in desmin mRNA expression was higher compared to healthy subjects. Intriguingly, the pattern of disorganized desmin often resembled the disorganization of mitochondria reported in the soft palate muscles of OSA patients [32] and in desmin mutations [33]. Given the suggested role of desmin in mitochondrial function and anchoring [34], this disorganization could potentially affect energy metabolism and myofibrillar function in the upper airway muscles of OSA patients.

The presence of intracellular aggregates containing both desmin protein and its corresponding mRNA in OSA patients provides additional evidence of muscle impairment [3]. Protein aggregation in myofibrillar myopathies is a specific and significant phenomenon affecting myofiber function by disrupting the sarcomere architecture [35]. The misfolding of protein can happen due to errors in transcription or translation, mutations in the gene sequence, dysfunction of chaperones, and ubiquitin proteosome degradation pathways responsible for protein removal [36, 37]. Since reorganization and aggregates of desmin and its mRNA were not found in healthy subjects, traumatic snoring vibrations seem to be a strong candidate for the cause of pathological alterations in the upper airways of OSA patients [2, 3, 13, 16, 38, 39]. This theory is based on the fact that exposure to vibrating equipment can lead to nerve and muscle injuries [40]. Thus, central and peripheral predisposing factors reducing upper airway lumen may initiate harmful snoring vibrations leading to local neuromuscular injuries and muscle weakness. This might result in a vicious circle of increased traumatic snoring vibrations and neuromuscular damage contributing to swallowing dysfunction and upper airway collapse during sleep when the muscle tonus decreases [13, 41].

Muscle fiber injuries are commonly accompanied by fiber degeneration, followed by regeneration and repair [42]. Newly synthesized proteins are particularly susceptible to misfolding events [43, 44]. Thus, traumatic vibrations may not only cause muscle injuries but also induce disorganization and aggregates of desmin during the regeneration and repair of damaged myofibers [2, 45]. Moreover, it has been reported that desmin is upregulated during the process of muscle fiber regeneration after damage [46, 47]. This could explain the high expression of desmin mRNA observed in the muscle fibers we judged to be under regeneration. Interestingly, a population of muscle fibers from OSA patients exhibited extensive expression of desmin mRNA but lacked immunoexpression for the desmin protein. Disturbances in the translation process in OSA patients may hinder protein-coding or result in errors in amino acid composition, leading to reduced or non-functional proteins. This, in turn, could trigger a compensatory upregulation of desmin transcripts [48] explaining the overall increased expression of desmin mRNA as revealed by RT–qPCR.

## Conclusions

Our findings provide compelling evidence that a subgroup of muscle fibers lack both desmin mRNA and desmin protein within the human soft palate. This further strengthens the presence of a distinct subset of muscle fibers with a unique cytoskeletal structure, potentially indicating the existence of a novel fiber phenotype. These findings also show that desmin may be ubiquitous in all human muscles but not in all muscle fibers. Muscle fibers with unique cytoarchitecture might represent a specialization to meet the complex demands of various oropharyngeal functions. Furthermore, the presence of maldistribution or aggregation of the desmin protein and its mRNA in fibers of OSA patients indicates that the transcription and translation processes in the myofibers are disturbed. A likely cause for these alterations is long-term traumatic snoring vibrations.

## Data Availability

The data are available from the corresponding author upon reasonable request.

## Abbreviations

OSA: Obstructive sleep apnea
BMI: Mean body mass index
AHI: Apnea–hypopnea index
mRNA: Messenger ribonucleic acid
RT–qPCR: Reverse transcription-quantitative polymerase chain reaction
IF: Intermediate filament
H&E: Hematoxylin–eosin
pAb: Polyclonal antibody
mAb: Monoclonal antibody
DES: Desmin protein
PBS: Phosphate‐buffered saline
FITC: Fluorescein isothiocyanate
DAPI 4′: 6-Diamidino-2-phenylindole dihydrochloride
cDNA: Complementary deoxyribonucleic acid
DGC: Dystrophin–glycoprotein complex
